# From Single-Cell and Bulk Transcriptomic Integration to Functional Verification: Triaptosis-Associated lncRNA Signature Predicts Survival and Guides Therapy in Hepatocellular Carcinoma

**DOI:** 10.3390/ph18111691

**Published:** 2025-11-07

**Authors:** Xiaolong Liu, Ziyun Zhuang, Jiaxi Cheng, Yujie Li, Duguang Li, Zhaoqi Shi, Jing Yang, Xiaoxiao Fan, Hui Lin

**Affiliations:** 1 Department of General Surgery, Sir Run Run Shaw Hospital, School of Medicine, Zhejiang University, Hangzhou 310016, China; 12218318@zju.edu.cn (X.L.);; 2 School of Medicine, Shantou University Medical College, Shantou 515000, China

**Keywords:** triaptosis, hepatocellular carcinoma, lncRNA, prognostic signature, single-cell omics

## Abstract

**Background:** Hepatocellular carcinoma (HCC) continues to be a major cause of cancer associated deaths worldwide, highlighting the need for new prognostic biomarkers and treatment strategies. Triaptosis, a recently characterized mode of regulated cell death, has shown potential as a therapeutic target in various malignancies, including HCC. Nevertheless, how long non-coding RNAs (lncRNAs) regulate triaptosis, as well as their function in HCC, is still not well understood. **Methods:** This study integrates bioinformatics and functional validation to delineate the interplay between lncRNAs and triaptosis in HCC progression. **Results:** Firstly, we confirm that pharmacologically inducing triaptosis, a process centrally mediated by ROS accumulation, with menadione sodium bisulfite (MSB) can inhibit HCC growth both in vitro and in vivo. Furthermore, single-cell RNA sequencing identifies a specific elevation of the triaptosis-related gene *MTM1* in malignant hepatocytes. Through systematic bioinformatics analysis of TCGA data, we develop a 5-lncRNA prognostic signature (*LINC01134*, *HPN-AS1*, *DDX11-AS1*, *AC009283.1*, *AC009005.1*) with superior predictive power over conventional clinical parameters. Strikingly, functional studies reveal that *LINC01134* acts as a crucial oncogenic driver and its depletion suppresses proliferation, migration, and invasion while sensitizing cells to triaptosis via *MTM1*-mediated PI(3)P catabolism. **Conclusions:** Collectively, our study confirms that triaptosis is a therapeutically targetable signaling in HCC and proposes *LINC01134* as a biomarker and therapeutic target, offering new insights into lncRNA-mediated regulation of cell death for precision oncology.

## 1. Introduction

HCC represents a complex and globally significant malignancy [1]. Its early-stage clinical symptoms are often subtle, coupled with diverse risk factors, making early diagnosis and management particularly challenging. According to the 2024 global cancer statistics, primary liver cancer is the sixth most commonly diagnosed cancer and the third most common cause of cancer death worldwide [2]. Among primary liver cancers, HCC constitutes 80–90% of cases, standing as the predominant subtype [3]. Recent advances in targeted therapies and immunotherapies have provided promising avenues for improving patient survival [4]. Nevertheless, HCC remains associated with high recurrence rates, metastatic potential, and therapeutic resistance, contributing to persistently poor prognoses [5]. For instance, a significant number of advanced HCC patients derive limited long-term benefits from systemic therapies because of inherent or acquired drug resistance. Coupled with the increasing global incidence of HCC, this resistance contributes to the persistently high mortality rates associated with the disease [6]. These persistent obstacles underscore the dual imperative of advancing early diagnostic methods while accelerating the development of breakthrough treatment modalities against this aggressive malignancy.

Apoptotic and other regulated cell death mechanisms play fundamental roles in morphogenesis and tissue homeostasis, with their dysregulation being implicated in diverse pathologies ranging from malignancies to degenerative conditions [7]. A recent study has revealed that new epigenetic pathways and the 5-methylcytosine methylation of *MALAT1* contributed to sorafenib resistance in HCC, while simultaneously demonstrating the therapeutic potential of ferroptosis activation in overcoming this treatment resistance [6]. In addition, other studies have also established *YAP/TAZ* as critical negative regulators of ferroptosis that promote Sorafenib resistance in HCC [8]. Therefore, epigenetic modification of cell death can be a promising strategy for HCC therapy.

Recently, researchers investigated the therapeutic potential of pro-oxidant approaches for prostate cancer intervention. Their work identified dietary MSB—a mammalian vitamin K precursor derived from plant-based vitamin K (phylloquinone)—as a promising candidate. MSB functions as an oxidative inhibitor of PI 3-kinase VPS34, the key enzyme responsible for phosphatidylinositol-3-phosphate (PI(3)P) production. In prostate cancer models, MSB-induced oxidative stress significantly reduces PI(3)P levels, triggering a novel form of endosome-mediated cell death termed triaptosis [9]. This newly characterized cell death mechanism represents a breakthrough in cancer therapeutics, offering substantial potential for developing precision treatments targeting tumor-specific vulnerabilities.

Building on the exciting discovery of the critical role of epigenetic modifications in regulating cell death during HCC therapy, we also aim to investigate the potential involvement of epigenetic regulation in triaptosis in HCC. Among various epigenetic modifications, lncRNAs—defined as RNA transcripts longer than 200 nucleotides—are notable for their inability to encode proteins due to missing or non-functional open reading frames. Initially, this feature caused them to be dismissed as transcriptional noise rather than biologically relevant molecules [10]. The regulatory functions of long non-coding RNAs in cell death mechanisms represent a subject of intensive investigation within the field of hepatocellular carcinoma research [11]. For instance, emerging research has identified specific long non-coding RNAs, including *DUXAP8*, *URB1-AS1*, and *PVT1* as molecular mediators of sorafenib resistance through their regulatory roles in drug-induced ferroptosis pathways [12,13,14]. Previous studies have also shown that lncRNAs are closely associated with some programmed regulatory death mechanisms in HCC, such as cuproptosis and disulfidptosis [15].

This research utilized both in vitro and in vivo models to elucidate the role of triaptosis in HCC progression. A systematic identification and characterization of a set of triaptosis-related lncRNAs (TRLs) was undertaken utilizing data from the Cancer Genome Atlas (TCGA). Through integrated bioinformatics analysis incorporating both bulk and single-cell RNA sequencing data, we developed a robust 5-TRL prognostic signature that outperformed conventional clinical parameters in predicting patient outcomes. Our studies confirmed the potential of *LINC01134* as a therapeutic target, based on a series of in vitro and in vivo experiments. Collectively, these findings significantly advance our understanding of triaptosis-mediated molecular mechanisms in HCC and lay the groundwork for precision oncology strategies targeting triaptosis-regulatory networks.

## 2. Results

### 2.1. Triaptosis Induction Suppresses HCC Progression via ROS-Driven Cellular Stress

**Figure 1:**
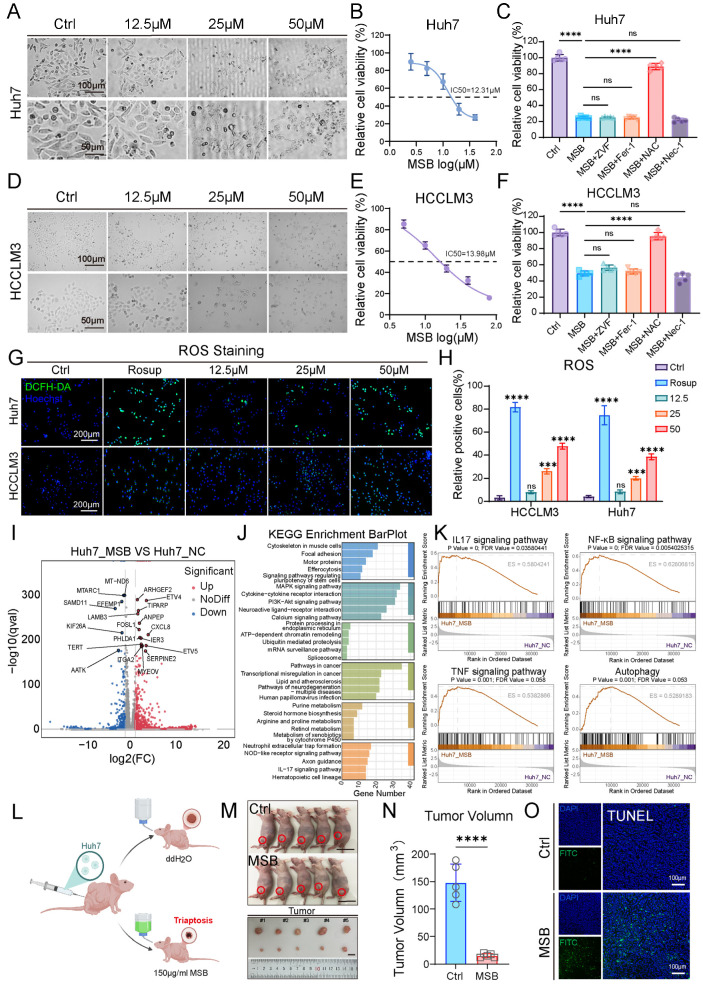
Triaptosis induction suppresses HCC progression through ROS-mediated cellular stress. (**A**,**D**) Morphological changes in Huh7 and HCCLM3 cells treated with escalating concentrations of MSB (0–50 μM) for 12 h, showing cytoplasmic vacuolization, membrane rupture, and dose-dependent cell death (scale bars: 100 μm, 50 μm). (**B**,**E**) Dose–response curves and half-maximal inhibitory concentrations (IC50: Huh7 = 12.31 μM; HCCLM3 = 13.98 μM). (**C**,**F**) Rescue of cell viability by co-treating with reactive oxygen species (ROS) scavenger *N*-acetyl-L-cysteine (NAC), but not other inhibitors: Z-VAD-FMK (ZVF, pan-caspase inhibitor), ferrostatin-1 (Fer-1, ferroptosis inhibitor), or necrostatin-1 (Nec-1, necroptosis inhibitor). (**G**) Intracellular ROS accumulation (green fluorescence) proportional to MSB concentration (scale bars: 200 μm; blue fluorescence: Hoechst). (**H**) Quantitative analysis of ROS levels by DCFH-DA fluorescence intensity. (**I**) Volcano plot of transcriptomic profiling in MSB-treated Huh7 cells (1159 upregulated [red] genes; 382 downregulated [blue] genes; |log_2_FC| > 1, *p* < 0.05). (**J**) KEGG pathway enrichment of upregulated genes highlighting inflammatory and autophagy pathways. (**K**) GSEA showing activation of IL-17, NF-κB, TNF signaling, and autophagy-associated cell death. (**L**) Schematic of subcutaneous xenograft model with Huh7 cells. (**M**) Representative images of tumors from control and MSB-treated mice. (**N**) Significant tumor volume reduction in MSB-treated mice vs. controls (**** *p* < 0.0001). (**O**) TUNEL staining confirming triaptosis-associated cell death in MSB-treated tumors (scale bars: 50 μm). Data: mean ± SD; statistics: Student’s *t*-test (two-group) or one-way ANOVA (multi-group); *** *p* < 0.001, **** *p* < 0.0001; ns: not significant.

To elucidate the regulatory role of triaptosis, a newly identified form of programmed cell death, in HCC progression, we first conducted in vitro experiments using the HCC cell lines Huh7 and HCCLM3. Cells were treated with escalating concentrations (0, 12.5, 25, 50 μM) of MSB, a known triaptosis inducer, for 12 h. A dose-dependent increase in cytoplasmic vacuolization, cellular swelling, and membrane rupture was observed (Figure 1A,D). The half-maximal inhibitory concentrations (IC50) were determined to be 12.31 μM for Huh7 and 13.98 μM for HCCLM3 (Figure 1B,E). To investigate the underlying mechanism of cell death, Huh7 and HCCLM3 cells treated with 25 μM MSB were co-treated with various cell death pathway inhibitors. Notably, only N-acetyl-L-cysteine (NAC), a reactive oxygen species (ROS) scavenger, significantly rescued cell viability (Figure 1C,F). Concurrently, intracellular ROS levels increased proportionally with MSB concentrations (Figure 1G,H), implicating ROS accumulation as a central driver of triaptosis in HCC cells.

Transcriptomic analysis of MSB-treated Huh7 cells identified 1159 upregulated and 382 downregulated genes (Figure 1I). Principal component analysis (PCA) clearly distinguished the MSB-treated group from the NCs, indicating distinct transcriptomic profiles (Figure S1A). Consistent with this, a heatmap of the top 100 most differentially expressed genes further illustrated the pronounced changes induced by MSB treatment (Figure S1B). KEGG and GSEA enrichment analyses highlighted the significant activation of inflammatory signaling pathways, including IL-17, NF-κB, and TNF, along with autophagy-associated cell death pathways (Figure 1J,K). These findings suggest that MSB disrupts cellular homeostasis and activates both inflammatory and pro-death cascades. Further focusing on ROS pathway-related genes, we observed that several key regulators—such as *CYP1A1*, *VEGFA*, *JUN*, and *FOXO3*—were significantly overexpressed in MSB-treated Huh7 cells (Figure S1C), supporting a link between MSB treatment and oxidative stress response.

To validate the in vivo relevance of triaptosis, we developed a xenograft model by subcutaneously inoculating nude mice with Huh7 cells (Figure 1L). Mice in the treatment group received 150 μg/mL MSB in drinking water for two weeks. Compared to controls, MSB-treated mice exhibited a significant reduction in tumor volume (*p* < 0.0001; Figure 1M,N), highlighting the therapeutic potential of triaptosis induction in suppressing HCC progression. Analysis of tumor tissue TUNEL staining revealed extensive cell death in MSB-treated tumors and confirmed the induction of triaptosis in murine HCC (Figure 1O).

### 2.2. Transcriptomic Analysis of Triaptosis-Associated Genes at Single-Cell Resolution in Human HCC and Matched Normal Liver

**Figure 2:**
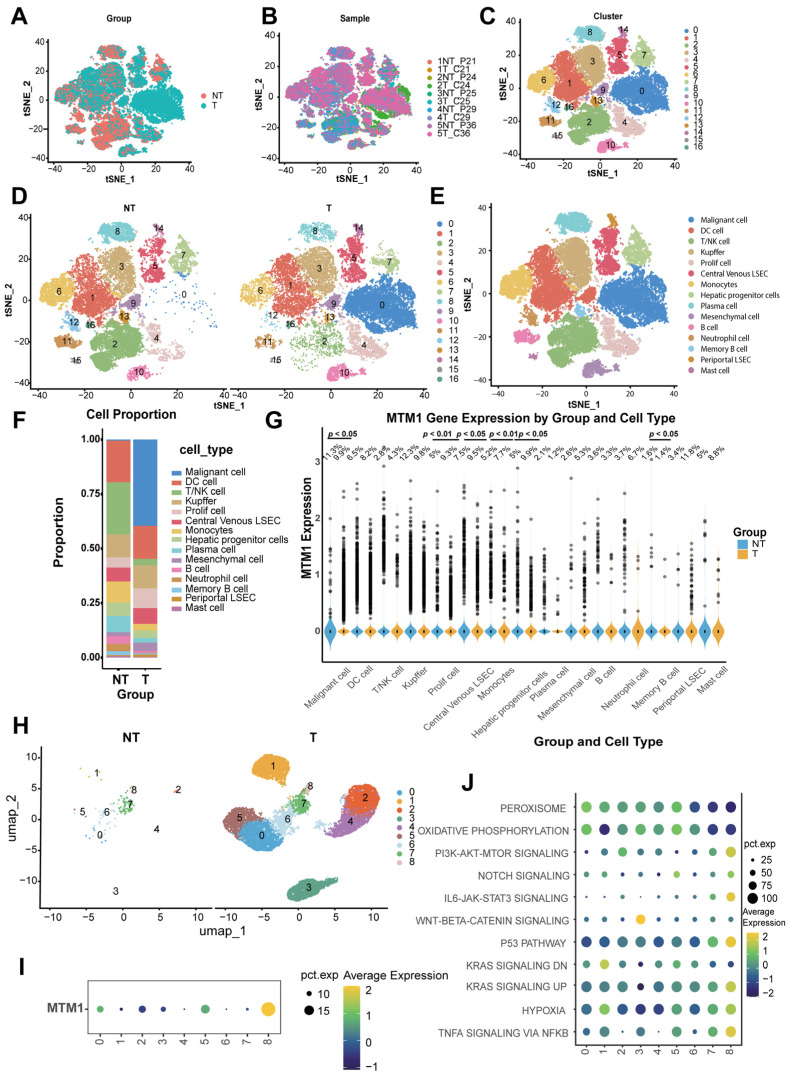
Single-cell transcriptomic landscape reveals *MTM1* dysregulation in HCC. (**A**) t-SNE visualization of 58,845 high-quality single cells profiled from paired HCC tumors (n = 5) and matched normal liver tissues (n = 5) using 10X Genomics scRNA-seq (GSE242889). (**B**) Batch-corrected t-SNE projection stratified by patient origin, demonstrating minimal technical batch effects. (**C**) Unsupervised clustering analysis resolving 17 transcriptionally distinct cellular subgroups across the integrated dataset. (**D**) Subgroup distribution between HCC and matched normal tissues. (**E**) t-SNE plots annotated by cell types in combined cohorts. (**F**) Proportional composition of 15 cell types in HCC versus normal tissues. (**G**) Differential expression of the triaptosis-related gene *MTM1* across 15 cell types (tumor vs. normal). (**H**) High-resolution clustering of malignant hepatocytes identifies nine molecularly distinct neoplastic subpopulations. (**I**) Heterogeneous *MTM1* expression patterns across malignant hepatocyte subclusters. (**J**) Pathway activation heatmap reveals cluster 8-specific enrichment of oncogenic signaling networks (PI3K-AKT-mTOR, TNFα-NF-κB, p53, and KRAS pathways).

Validation of triaptosis in HCC patients was performed through analysis of single-cell RNA-sequence data from paired tumor–normal tissues of five treatment-naïve individuals (Figure 2A,B). Following stringent quality control to exclude low-quality cells, we retained 58,845 high-confidence single cells for downstream analysis (Figure S2A–F).

Cell type annotation was achieved through unsupervised clustering with iterative refinement of resolution parameters, identifying stable clustering hierarchies at resolutions of 0.1–0.3 (Figure S2G). Application to the integrated dataset resolved 17 distinct clusters (Figure 2C,D), annotated using canonical marker genes (Figure S2H). t-SNE visualization further delineated 15 major cell populations (Figure 2E), including malignant hepatocytes, dendritic cells (DCs), T/NK cells, Kupffer cells, proliferating cells, central venous liver sinusoidal endothelial cells (LSECs), monocytes, hepatic progenitor cells, plasma cells, B cells, neutrophils, memory B cells, periportal LSECs, and mast cells (signature genes detailed in Supplementary Table S1).

Comparative analysis highlighted pronounced differences in cellular composition between tumor and normal tissues (Figure 2F), with malignant hepatocytes significantly enriched in HCC samples, aligning with their tumorigenic role. Strikingly, *MTM1*—a core triaptosis-associated gene—showed elevated expression in malignant cells from tumor tissues (Figure 2G). This tumor-specific upregulation suggests a potential therapeutic vulnerability, where pharmacological induction of triaptosis could selectively target HCC cells.

To comprehensively delineate the intratumoral heterogeneity of malignant hepatocytes, we performed high-resolution transcriptomic mapping that delineated nine molecularly distinct hepatocellular subclusters (Figure 2H). Notably, cluster 8 emerged as a unique subpopulation exhibiting selective enrichment of *MTM1* expression (Figure 2I), the core triaptosis-associated gene. Functional pathway profiling using cancer hallmark signatures revealed differential activation patterns across subclusters: both PI3K-AKT-mTOR signaling and TNF-α–NF-κB axis activation were markedly amplified in cluster 8 (Figure 2J). Additionally, this aggressive subcluster showed concurrent activation of P53 signaling pathway and KRAS signaling pathway (Figure 2J). This synergistic hyperactivation of proliferative and inflammatory pathways aligns with clinically aggressive tumor phenotypes, suggesting that *MTM1*-enriched malignant cells may represent a therapeutically targetable subpopulation primed for triaptosis induction.

### 2.3. Identification of Triaptosis-Associated Differentially Expressed and Prognostic Long Non-Coding RNAs in HCC

**Figure 3:**
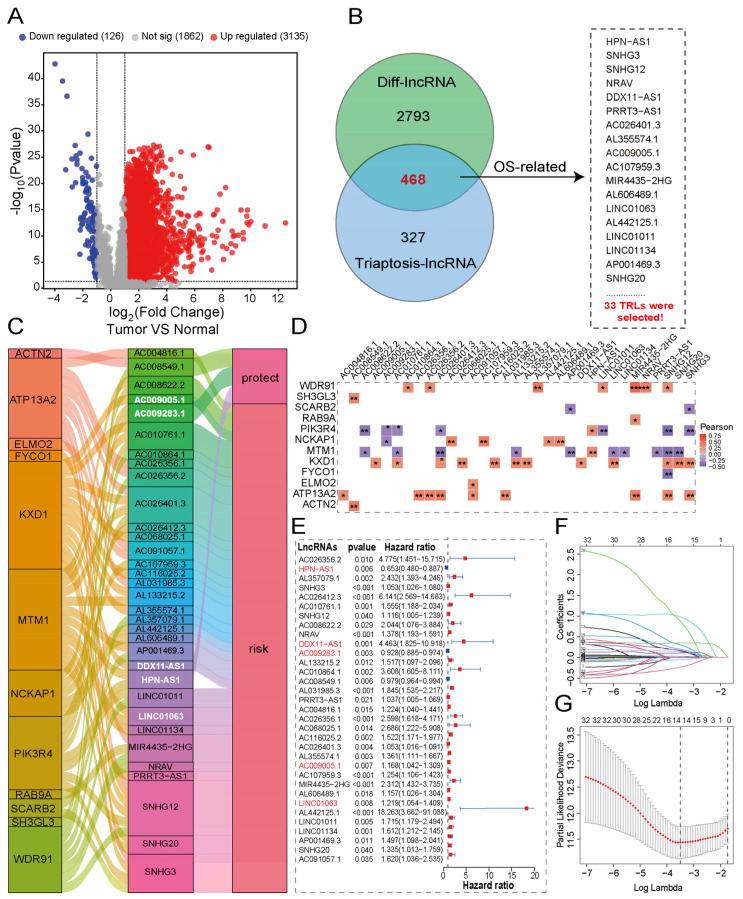
Construction of a triaptosis-associated lncRNAs prognostic signature in HCC. (**A**) Volcano plot of differentially expressed lncRNAs between HCC and normal tissues. (**B**) Venn diagram identifies 33 triaptosis-associated, prognostic, and differentially expressed lncRNAs (TADELs). (**C**) The Sankey diagram links TADELs with triaptosis-related genes (TRGs) via Pearson correlation (R > 0.3, *p* < 0.001). (**D**) Correlation heatmap of 33 TADELs with 12 TRGs in the TCGA-HCC cohort (red: positive; blue: negative). (**E**) Forest plot of univariate Cox regression results for the 33 TADELs. (**F**,**G**) LASSO coefficient profiles (**F**) and cross-validation error curves (**G**) for signature optimization. Statistical significance: * *p* < 0.05, ** *p* < 0.01, *** *p* < 0.001.

Initial analysis included 374 HCC patients from TCGA, from which we identified twelve triaptosis-related genes (TRGs) based on prior reports [9]. The study design flowchart is presented in the Graphical Abstract. To evaluate clinical significance, we performed comparative transcriptomic profiling between HCC tissues and matched adjacent normal tissues. Using stringent criteria (|log2FC| > 1, *p* < 0.001), we identified 3261 differentially expressed lncRNAs (DELs), with detailed annotations provided in Supplementary Table S2. The global expression landscape of lncRNAs in HCC versus normal tissues is depicted in Figure 3A through a volcano plot. The association between TRGs and lncRNAs was investigated by Pearson correlation analysis using stringent thresholds (|R| > 0.3, *p* < 0.001). This analysis identified 795 significantly correlated lncRNAs, as shown in Supplementary Table S3. Through integration of TRG-correlated lncRNAs (TCLs) and DELs, we established a novel category of 468 transcripts designated as Triaptosis-Correlated Differentially Expressed lncRNAs (TCDELs) (Figure 3B). Subsequent univariate Cox regression analysis of overall survival (OS) data from TCGA revealed 33 TCDELs with significant prognostic value (Figure 3B,E; Supplementary Table S4).

To comprehensively characterize the functional relationships between TCDELs and DRGs in HCC, we employed a Sankey diagram (Figure 3C) to visualize their intricate associations and prognostic relevance. This representation effectively demonstrates the network architecture linking molecular features with clinical outcomes in HCC patients. Correlation analysis delineated the relationships between prognostic TCDELs and TRGs (Figure 3D), where red and blue colors indicate positive (r > 0) and negative (r < 0) correlations, respectively. (Figure 3D). The relationship between TCDELs and the risk of HCC is shown in Figure 3E.

### 2.4. Developing and Validating a Triaptosis-Related lncRNA Signature for Prognostic Prediction in Hepatocellular Carcinoma

To quantify the prognostic utility of TCDELs, we developed a predictive signature through rigorous computational modeling. The cohort of 342 patients was randomly split into training and validation sets at a 1:1 ratio to ensure the model robustness. Based on the expression profiles of 33 candidate prognostic TCDELs, we applied LASSO regression (with the optimal penalty parameter λ selected by tenfold cross-validation as shown in Figure 3G) followed by multivariate Cox proportional hazards regression to construct the prognostic signature (Figure 3F). This process refined the model to five key triaptosis-related lncRNAs (TRLs), each demonstrating independent predictive value. These five TRLs were incorporated into the final signature, with their relative contributions weighted by the corresponding regression coefficients. The risk score for each HCC patient was calculated using the following formula: Risk Score = (0.6582 × *LINC01134* expression) + (−0.5398 × *HPN-AS1* expression) + (1.8022 × *DDX11-AS1* expression) + (−0.8078 × *AC009283.1* expression) + (0.368 × *AC009005.1* expression). Detailed information is provided in Supplementary Table S5.

**Figure 4:**
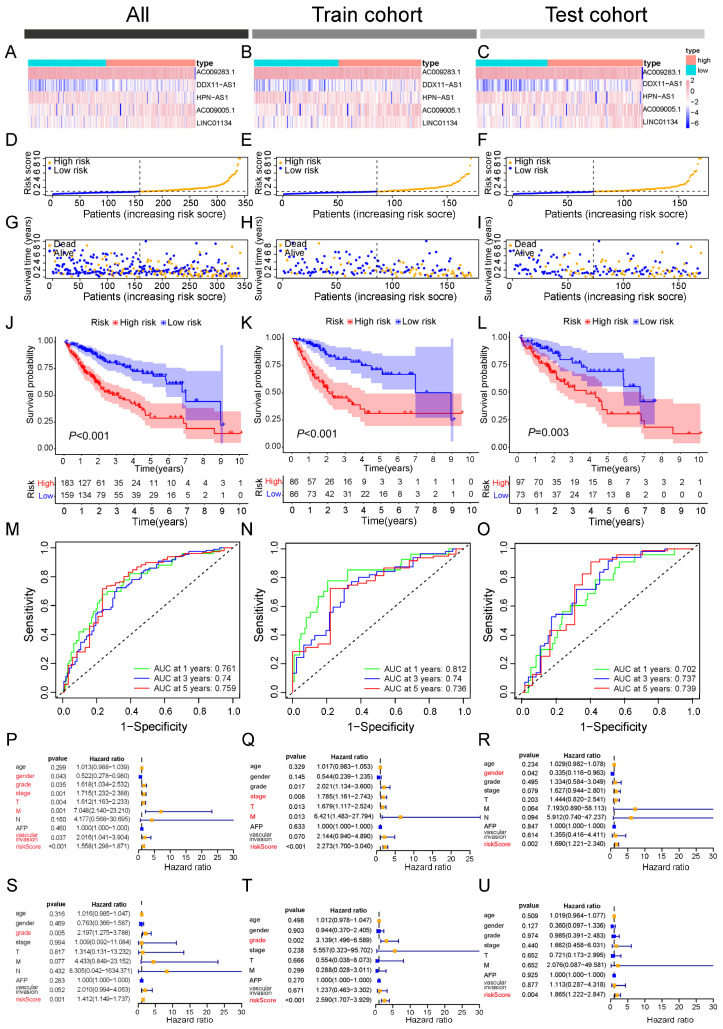
Validation of the triaptosis-related lncRNA (TRL) prognostic model in HCC cohorts. (**A**–**C**) Risk score distributions in the (**A**) entire cohort, (**B**) training cohort, and (**C**) validation cohort. (**D**–**F**) Median risk scores across cohorts. (**G**–**I**) Survival status, survival time, and risk score alignment in each cohort. (**J**–**L**) Kaplan–Meier survival curves for high- versus low-risk subgroups (overall, training, and validation cohorts). (**M**–**O**) Time-dependent receiver operating characteristic (ROC) curves evaluating 1-, 3-, and 5-year overall survival (OS) prediction (AUC values shown). Data presented as mean ± SD; log-rank test for survival analysis. (**P**–**R**) The univariate Cox regression analyses of overall, training, and validation cohorts. (**S**–**U**) The multivariate Cox regression analyses of overall, training, and validation cohorts.

Patients were stratified into low- and high-risk subgroups based on the median risk score. And the expression patterns of the five-TRL signature across these subgroups are displayed in the heatmap (Figure 4A–C). Both Principal Component Analysis (PCA) and t-Distributed Stochastic Neighbor Embedding (t-SNE) confirmed clear separations between the two risk subgroups in the training, testing, and overall cohorts (Figure S3A–F). To evaluate the model’s robustness and clinical applicability, we assessed survival outcomes across all cohorts. The results consistently demonstrated that high-risk patients experienced significantly higher mortality, whereas low-risk patients were associated with more favorable survival outcomes (Figure 4D–I).

Kaplan–Meier survival curves further confirmed the prognostic value of the risk stratification, showing significantly poorer overall survival in high-risk patients across all cohorts (log-rank *p* < 0.05; Figure 4J–L). The predictive performance of the signature was evaluated using time-dependent ROC analysis, which revealed strong discriminative ability for 1-, 3-, and 5-year survival in the entire cohort (AUCs = 0.761, 0.740, and 0.750, respectively). Similar predictive accuracy was maintained in the training (AUCs = 0.812, 0.740, and 0.736) and testing cohorts (AUCs = 0.702, 0.737, and 0.739; Figure 4M–O).

To assess whether the risk score represents an independent prognostic factor in HCC, univariate and multivariate Cox regression analyses were conducted. Univariate analysis indicated that the risk score was significantly associated with adverse outcomes (HR = 1.558, 95% CI = 1.298–1.871, *p* < 0.001), along with gender, tumor grade, clinical stage, T stage, M stage, and vascular invasion (Figure 4P). In the multivariate model, however, only tumor grade (HR = 2.197, 95% CI = 1.275–3.788, *p* < 0.005) and the risk score (HR = 1.412, 95% CI = 1.149–1.737, *p* < 0.001) retained independent prognostic value (Figure 4S). Consistent results were observed in both training and testing cohorts, where the risk score outperformed other clinical variables as an independent predictor (Figure 4Q–U).

Taken together, these results confirm the TRL-based signature as a robust and independent prognostic indicator for HCC patients.

### 2.5. Association of the TRL Signature with Clinicopathological Characteristics and Development of a Prognostic Nomogram in HCC Patients

**Figure 5:**
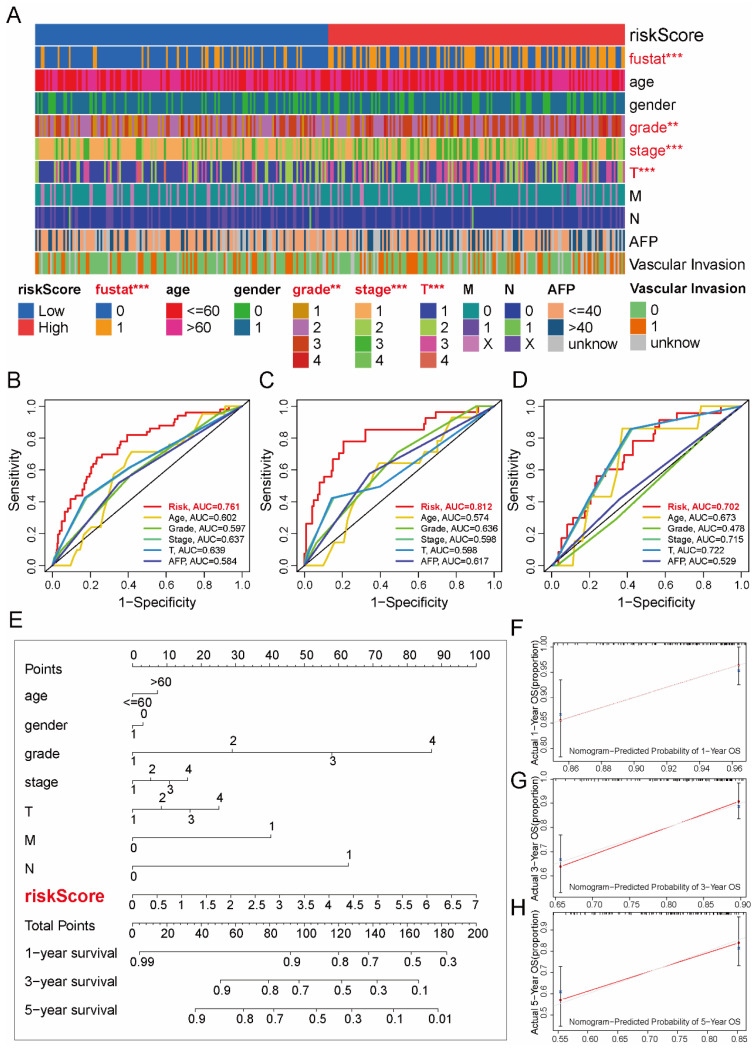
Clinical relevance and prognostic nomogram integrating the TRL signature in HCC. (**A**) Heatmap of clinicopathological features (red: significant differences, *p* < 0.05) stratified by TRL risk subgroups. (**B**–**D**) ROC curves comparing TRL signature performance against conventional prognostic markers in the entire (**B**), training (**C**), and validation (**D**) cohorts. (**E**) Multivariable nomogram combining TRL risk scores with clinical variables (age, sex, grade, TNM stage, vascular invasion) for OS prediction. (**F**–**H**) Calibration plots for 1-, 3-, and 5-year OS probabilities (observed vs. nomogram-predicted). Statistical significance: ** *p* < 0.01, *** *p* < 0.001; AUC, area under the curve.

We assessed the clinical relevance of the TRL risk stratification by examining its correlations with key clinicopathological features (Figure 5A). The analysis revealed statistically significant associations between risk groups and several parameters, including survival status (*p* < 0.001), histological grade (*p* < 0.01), T stage (*p* < 0.001), and overall pathological stage (*p* < 0.001). The prognostic discriminative power of the 5-TRL signature was then rigorously validated through ROC curve analysis. Comparative evaluation against conventional clinicopathological indicators (Figure 5B–D) revealed superior predictive accuracy, with AUC values of 0.761 (entire cohort), 0.812 (training set), and 0.702 (validation set), significantly outperforming all individual clinical parameters in predictive accuracy.

For enhanced clinical applicability, we constructed a multivariate prognostic nomogram integrating the TRL risk signature with critical clinical variables: age, gender, histological grade, TNM stage (T, N, M), and overall pathological stage (Figure 5E). At 1-, 3-, and 5-year time points, the calibration curves revealed that survival probabilities predicted by the nomogram closely matched the observed outcomes (Figure 5F–H). The proximity to the 45° reference line demonstrates a high degree of concordance, supporting the model’s clinical reliability.

### 2.6. Clinical Validation and Prognostic Significance of LINC01134 in HCC

A total of 16 paired tumor and adjacent normal tissues were obtained from HCC patients undergoing surgical resection to systematically evaluate the clinical relevance of candidate lncRNAs. Subsequent qRT-PCR validation demonstrated that among five lncRNAs comprising our prognostic signature, *LINC01134* displayed the most pronounced differential expression between malignant and non-malignant tissues (*p* < 0.0001, Figure S4). This tumor-specific overexpression pattern implicated *LINC01134* as a potential molecular effector in HCC pathogenesis. Notably, given its association with triaptosis (Figure 3C,D), we postulated that *LINC01134* might functionally interconnect triaptosis pathways and HCC progression.

**Figure 6:**
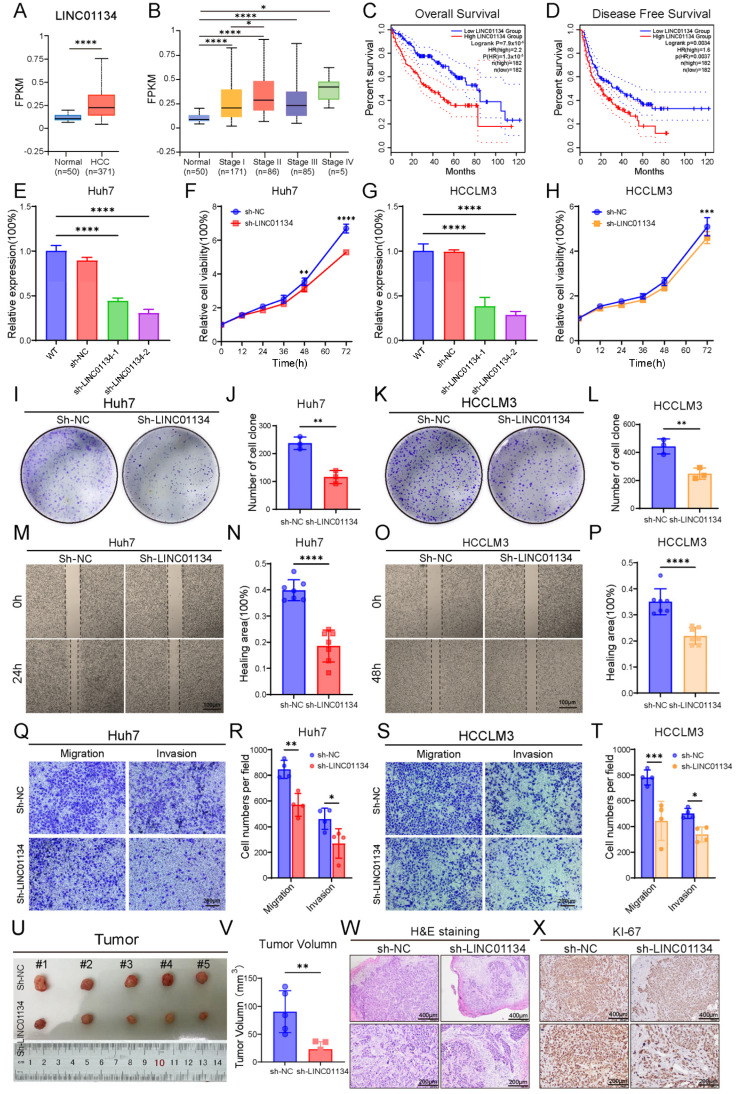
Clinical and functional characterization of *LINC01134* in HCC. (**A**) Analysis of TCGA-LIHC data reveals significant upregulation of *LINC01134* in HCC tissues compared to normal liver tissues. (**B**) *LINC01134* expression increases with tumor progression (AJCC staging). (**C**,**D**) Kaplan–Meier survival analysis demonstrates that high *LINC01134* expression correlates with worse OS (log-rank test, *p* < 0.0001) and DFS (log-rank test, *p* = 0.0038). (**E**,**G**) Efficient *LINC01134* knockdown in Huh7 and HCCLM3 cells using shRNA-1 and shRNA-2 (*p* < 0.0001 vs. NC). (**F**,**H**) CCK-8 assays demonstrate significantly reduced proliferation in *LINC01134*-silenced cells over 72 h. (**I**,**K**) Representative images of colony formation assays showing decreased clonogenicity in *LINC01134*-silenced cells. (**J**,**L**) Quantitative analysis of cell colony numbers in colony formation assays. (**M**,**O**) Wound healing assays of Huh7 and HCCLM3 cells at 0, 24, and 48 h. (**N**,**P**) Quantitative analysis of wound closure rates. (**Q**,**S**) Transwell migration and invasion assays of Huh7 and HCCLM3 cells. (**R**,**T**) Quantification of migrated and invaded cells. (**U**) Representative photographs of subcutaneous tumors from NC and sh-*LINC01134* groups on day 14. (**V**) Quantification of tumor growth volumes. (**W**) H&E staining reveals disrupted tumor architecture and increased fibrosis in sh-*LINC01134* xenografts. (**X**) Decreased Ki-67-positive cells in sh-*LINC01134* tumors by IHC. Data are presented as mean ± SD. Statistical significance was determined by Student’s *t*-test or one-way ANOVA: * *p* < 0.05, ** *p* < 0.01, *** *p* < 0.001, **** *p* < 0.0001.

To substantiate this hypothesis, we initially analyzed *LINC01134* expression in the TCGA-LIHC dataset. The bioinformatic results aligned with our clinical observations, demonstrating significant *LINC01134* upregulation in HCC tissues compared to normal controls (*p* < 0.0001, Figure 6A). Importantly, a progressive elevation of *LINC01134* expression was observed across advancing tumor stages (AJCC classification), suggesting its potential role in disease progression (Figure 6B). Using median *LINC01134* expression as cutoff, patients were divided into high- and low-expression groups (n = 182 each). The high-expression group exhibited significantly shorter overall (*p* < 0.0001) and disease-free survival (*p* = 0.0034) (Figure 6C,D), supporting *LINC01134*’s role as an independent prognostic factor in HCC.

### 2.7. Functional Characterization of LINC01134 in HCC Progression

To mechanistically dissect the oncogenic role of *LINC01134*, we generated stable *LINC01134*-knockdown HCC cell lines (Huh7 and HCCLM3) using a lentiviral shRNA system. qRT-PCR confirmed efficient silencing of *LINC01134* (>70%) with shRNA-2 (*p* < 0.0001 vs. control, Figure 6E,G). CCK-8 proliferation assays exhibited markedly reduced viability in *LINC01134*-silenced Huh7 (*p* < 0.0001) and HCCLM3 (*p* < 0.001) cells over 72 h (Figure 6F,H). Complementary colony formation assays further validated these findings, with both cell lines demonstrating >50% reduction in clonogenic capacity post-knockdown (*p* < 0.001, Figure 6I–L). We further evaluated the impact of *LINC01134* on metastatic potential. Wound-healing assays under serum-starved conditions demonstrated impaired migration in *LINC01134*-silenced Huh7 and HCCLM3 cells at 24/48 h post-wounding (*p* < 0.0001, Figure 6M–P). Similarly, transwell assays revealed reduced migratory and invasive capacities in both cell lines. Huh7 migration and invasion decreased by 30% (*p* < 0.01) and 40% (*p* < 0.05), respectively, while HCCLM3 exhibited 45% (*p* < 0.001) and 30% (*p* < 0.05) reductions (Figure 6Q–T). These results collectively established *LINC01134* as a critical driver of HCC proliferation and metastasis.

To investigate the tumorigenic role of *LINC01134* in a physiological context, we established a subcutaneous xenograft model. For this purpose, male BALB/c nude mice, aged six weeks, were utilized, with six mice assigned to each experimental group. Huh7 cells stably transfected with either control (NC) or *LINC01134*-specific shRNA (sh-*LINC01134*) was suspended in PBS and inoculated into the right flank (1 × 10^7^ cells per site). Tumor growth was monitored every 48 h using digital calipers, with volumes calculated according to the formula (length × width^2^)/2. At 14 days post-implantation, the animals were euthanized for endpoint analysis. Consistent with vitro observations, sh-*LINC01134* xenografts exhibited significantly impaired tumor growth compared to NCs, displaying an approximately 70% reduction in final tumor volume (*p* < 0.01, Figure 6U,V). Histopathological assessment of formalin-fixed paraffin-embedded (FFPE) sections revealed distinct morphological alterations: NC tumors consisted of densely packed neoplastic cells with high nuclear–cytoplasmic ratios, whereas sh-*LINC01134* tumors exhibited disorganized architecture, increased stromal fibrosis, and multifocal degenerative changes (Figure 6W). To assess proliferative activity, IHC staining for Ki-67, a well-established proliferation marker, was performed. The results demonstrated a marked reduction in Ki-67-positive nuclei in sh-*LINC01134* tumors compared to controls (Figure 6X).

Collectively, these findings indicate that genetic ablation of *LINC01134* not only attenuates HCC proliferation in vitro, but also substantially suppresses tumorigenesis in vivo, further supporting its classification as an oncogenic driver.

### 2.8. Functional and Mechanistic Insights into LINC01134-Mediated Triaptosis Regulation in HCC

**Figure 7:**
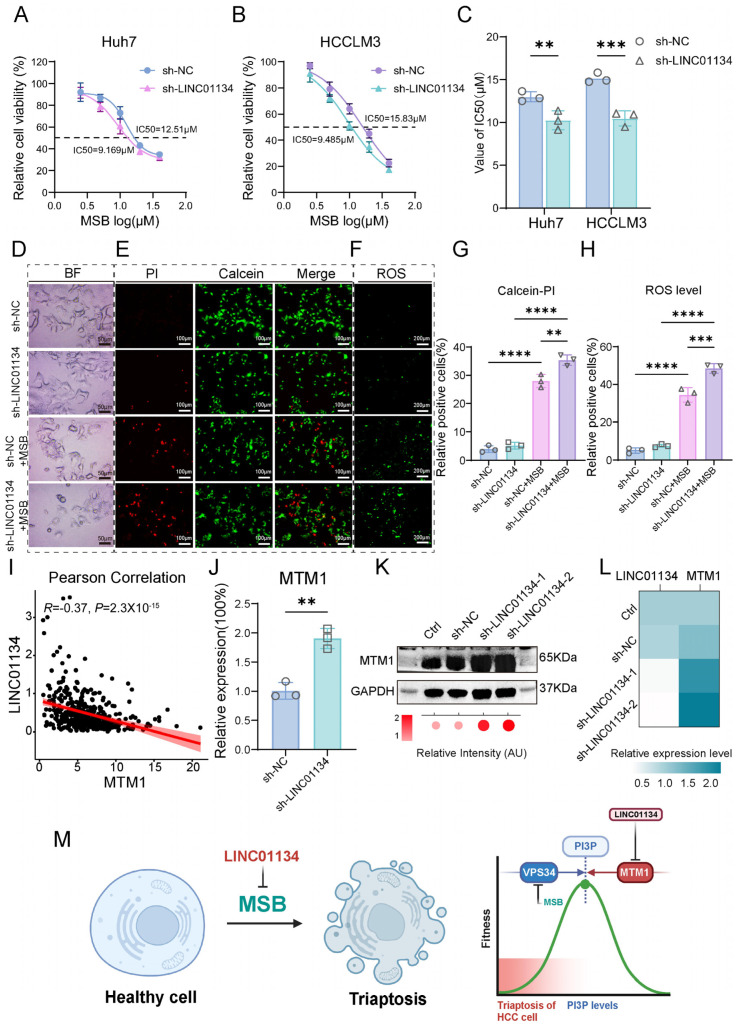
Functional and mechanistic insights into *LINC01134*-mediated triaptosis regulation in HCC. (**A**,**B**) Dose–response curves of Huh7 and HCCLM3 cells treated with MSB, showing reduced IC50 values in *LINC01134*-knockdown (sh-*LINC01134*) versus control (sh-NC) cells. (**C**) Statistical validation of triaptosis sensitivity across three biological replicates (Student’s *t*-test). (**D**) Bright-field microscopy images revealing accelerated cytoplasmic vacuolization and membrane rupture in sh-*LINC01134* cell post-MSB treatment (scale bar: 50 μm). (**E**,**G**) Calcein-AM/PI dual staining quantifying PI-positive (dead) cells in sh-*LINC01134* and sh-NC Huh7 cells (scale bar: 100 μm). (**F**,**H**) DCFH-DA staining demonstrating elevated ROS levels in sh-*LINC01134* Huh7 cells (scale bar: 200 μm). (**I**) Scatter plot showing negative correlation between *LINC01134* and *MTM1* mRNA expression in HCC cohorts (Pearson’s R = −0.37, *p* < 0.0001). (**J**–**L**) qRT-PCR and Western blot analyses of *MTM1* mRNA and protein levels in sh-*LINC01134* Huh7 cells (normalized to GAPDH). (**M**) Schematic model: *LINC01134* sustains PI(3)P pools by suppressing *MTM1*-mediated dephosphorylation, thereby desensitizing HCC cells to triaptosis. Data are presented as mean ± SD. Statistical significance was determined by Student’s *t*-test: ** *p* < 0.01, *** *p* < 0.001, **** *p* < 0.0001.

Building upon our earlier triaptosis-related prognostic model containing five lncRNAs, we prioritized *LINC01134*—a candidate oncogenic lncRNA whose tumor-promoting roles in HCC were validated through functional assays spanning in vitro proliferation/migration and in vivo xenograft growth. To elucidate its regulatory role in triaptosis, we engineered stable *LINC01134*-knockdown (sh-*LINC01134*) and control (sh-NC) HCC cell lines (Huh7 and HCCLM3) and subjected them to MSB. Strikingly, *LINC01134* depletion significantly enhanced triaptosis susceptibility, reducing IC50 values from 12.51 μM to 9.17 μM in Huh7 and 15.83 μM to 9.49 μM in HCCLM3 (Figure 7A,B), with statistical significance confirmed across three biological replicates (Huh7: *p* < 0.01; HCCLM3: *p* < 0.001; Figure 7C).

Morphological analysis revealed accelerated cytoplasmic vacuolization and cell deformation in sh-*LINC01134* cells post-MSB treatment (Figure 7D). Quantitative validation Via Calcein-AM/PI dual staining demonstrated a 26.6% increase in PI-positive (dead) cells in sh-*LINC01134* Huh7 compared to sh-NC (Figure 7E,G). Concurrently, ROS-specific staining (DCFH-DA) showed a 40.9% elevation in intracellular ROS levels in knockdown cells (Figure 7F,H), directly linking *LINC01134* loss to oxidative stress amplification. These findings establish *LINC01134* as a critical suppressor of triaptosis in HCC, with depletion potentiating cell death via ROS-driven metabolic collapse.

To delineate the mechanistic axis linking *LINC01134* to triaptosis regulation, we integrated genome-wide correlation analyses between *LINC01134* and triaptosis hallmark genes (Figure 3D). Strikingly, *LINC01134* exhibited a moderate but significant negative correlation with *MTM1* (myotubularin 1; R = −0.37, *p* < 0.0001), a dual-specificity phosphatase that dephosphorylates PI(3)P to PI, thereby antagonizing VPS34 kinase activity—a key node in triaptosis execution (Figure 7I). The increased sensitivity to triaptosis observed upon *LINC01134* depletion is mechanistically explained by the upregulation of *MTM1*. In Huh7 cells, knockdown of *LINC01134* significantly elevated *MTM1* levels, yielding a 1.9-fold increase in mRNA (*p* < 0.01) and a 2.1-fold increase in protein (*p* < 0.05) (Figure 7J–L). This rise in *MTM1* expression accounts for the heightened sensitivity, as it would consequently suppress the accumulation of PI(3)P, which is a necessary step for triaptosis occurrence.

We propose a model wherein *LINC01134* acts as a silencer of *MTM1*, sustaining PI(3)P pools by blocking its dephosphorylation. This desensitizes HCC cells to triaptosis, whereas *LINC01134* depletion unleashes *MTM1*-mediated PI(3)P catabolism, driving cell death (Figure 7M).

## 3. Discussion

This study provides comprehensive evidence linking triaptosis, a novel form of programmed cell death, to lncRNA-mediated regulatory networks in HCC. Our findings reveal that ROS-driven triaptosis induction effectively suppresses HCC progression, aligning with prior reports of as a vulnerability in HCC [16]. The integration of single-cell transcriptomics further uncovered *MTM1* upregulation in malignant hepatocytes, suggesting that triaptosis activation could selectively target tumor cells while sparing normal tissues—a critical advantage for therapeutic development. Central to this work is the identification of a 5-lncRNA prognostic signature with superior predictive accuracy over conventional clinical staging. Among these, *LINC01134* emerged as a multifaceted regulator, driving tumor aggressiveness while suppressing triaptosis sensitivity. Mechanistically, *LINC01134* silencing enhanced *MTM1* expression, thereby depleting PI(3)P-inducing triaptosis execution. This dual role positions *LINC01134* as both a prognostic biomarker and a therapeutic target. Notably, our results extend previous studies on lncRNA-mediated drug resistance by introducing a novel axis connecting lncRNAs to cell death regulation.

The five lncRNAs in our prognostic signature—*HPN-AS1*, *DDX11-AS1*, *LIN01134*, *AC009283.1*, and *AC009005.1*—exhibit potential roles in HCC progression and triaptosis regulation. Previous studies have implicated these lncRNAs in various cancers. For instance, *HPN-AS1* was demonstrated by Jin et al. to suppress HCC proliferation via *GABPA*-mediated transcriptional activation and *eIF4A3* degradation [17]. Our analysis aligns with these findings, as higher *HPN-AS1* expression was correlated with improved prognosis in low-risk HCC patients, suggesting its protective role in specific molecular subtypes. In contrast, *DDX11-AS1* exhibits oncogenic properties across malignancies, including bladder cancer, glioma, and HCC [18,19]. Li et al. linked its demethylation-driven overexpression to p53 degradation and impaired tumor suppressor signaling through the *PARP1/p53* axis, underpinning its association with aggressive disease [20]. Additionally, Miranda et al. suggested a causal link between *AC009283.1* and carcinogenesis, reporting that this lncRNA enhances tumor cell proliferation and apoptosis resistance in *HER2*-positive breast cancer [21]. Notably, *AC009005.1* emerges as a novel high-risk biomarker, with elevated expression predicting poor survival in HCC, yet its mechanistic contributions remain unexplored, highlighting an urgent need to delineate its regulatory pathways in HCC malignancy and triaptosis. The functional heterogeneity of these lncRNAs across cancer types highlights the critical need for context-specific validation. Future studies integrating multi-omics data (e.g., epigenomic, proteomic, and ubiquitinomic profiling) could elucidate their interplay with triaptosis and HCC and then inform targeted therapies leveraging epigenetic or post-translational modulation.

Emerging research has highlighted the critical role of lncRNAs in modulating tumor cell death mechanisms through multidimensional regulatory networks, underscoring their dynamic plasticity and functional diversity in cancer progression [22]. For instance, recent investigations by Lin’s group have elucidated distinct lncRNA-mediated paradigms: lncRNA *LncFASA* promotes ferroptosis in triple-negative breast cancer via *PRDX1* phase-separation-mediated lipid peroxide accumulation [23]. Separately, in colorectal cancer, the tumor-suppressive lncRNA *LINC00982* encodes *PRDM16-DT*, which interacts with *HNRNPA2B1* to suppress *CHEK2* exon skipping. This generates the L-CHEK2 splice variant, activating fibroblast-derived MMP9 secretion and thereby inhibiting tumor metastasis and chemoresistance [24]. Concurrently, Liu’s team uncovered a novel mechanism in gastric cancer where the lncRNA *HCP5* regulates ferroptosis. Overexpression of *HCP5* stabilizes ferroptosis-related genes via the *HCP5-132aa/YBX1/ELAVL1* ternary complex, suppressing ferroptosis and accelerating cancer progression. CRISPR/Cas9-mediated *HCP5* knockout enhanced sensitivity to ferroptosis inducers, suggesting therapeutic potential [25]. These findings collectively revealed lncRNA-mediated regulatory paradigms—including epigenetic modification, phase separation, alternative splicing, post-translational modification, and microprotein encoding—that shape tumor microenvironments and regulate death pathways [11,22].

These findings collectively revealed lncRNA-mediated regulatory paradigms—including epigenetic modification, phase separation, alternative splicing, post-translational modification, and microprotein encoding—that shape tumor microenvironments and regulate cell death pathways [26]. Consistent with our study, we found that *LINC01134* shown to promote HCC progression by suppressing sensitivity to triaptosis, a programmed cell death mechanism. This finding reinforces the role of lncRNAs as key regulators of cancer cell death sensitivity. Together, these insights provide a theoretical foundation for developing lncRNA-targeted nucleic acid therapies and combinatorial strategies using programmed cell death inducers. Future studies should prioritize deciphering the spatiotemporal interaction networks of lncRNAs to expedite their clinical translation.

The clinical value of our findings is underscored by several observations: (1) notably, the signature demonstrated stage-independent prognostic value across all TNM subgroups, highlighting its capacity to overcome the limitations of anatomic staging and provide molecular-driven risk assessment for precision oncology applications. (2) *LINC01134* expression correlated with advanced AJCC stages, reflecting its functional contribution to disease aggressiveness. (3) By integrating molecular signatures with conventional clinical parameters, the developed nomogram showed strong predictive accuracy and may serve as a practical tool for personalized prognostic assessment and therapeutic decision-making. (4) *LINC01134* silencing upregulated *MTM1* expression, thereby enhancing the sensitivity of HCC cells to triaptosis. This finding expands the potential for targeted tumor therapies through the induction of programmed cell death pathways.

However, certain limitations warrant consideration. First, while our study demonstrates that genetic ablation of *LINC01134* inhibits tumor progression in vivo and suppresses *MTM1* expression in vitro, it should be noted that the causal relationship between *LINC01134* knockout-mediated tumor suppression and *MTM1* downregulation remains to be formally established in animal models. Specifically, we have not yet provided direct evidence that *LINC01134* deletion reduces *MTM1* expression in vivo, and such reduction functionally contributes to the observed antitumor effects. Thirdly, while murine xenograft models validated *LINC01134*’s oncogenic role, orthotopic models better recapitulating the liver microenvironment are needed. Fourthly, another important limitation is the unexplored functional contribution of VPS34, a key modulator of triaptosis that directly antagonizes *MTM1* activity, in our experimental system.

For clinical translation, future studies should include the following: first, developing combination regimens integrating novel triaptosis activators (e.g., MSB derivatives) with *LINC01134* silencing approaches. Second, conducting phase II biomarker-driven trials to validate the signature’s predictive value. Third, systematically mapping death pathway among triaptosis and other cell death pathways interactions through CRISPR screens to design rational polytherapy regimens against resistant clones.

## 4. Material and Methods

### 4.1. External Data Collection

RNA-seq data (FPKM-standardized) from 374 HCC samples and 50 adjacent normal liver tissues were obtained from TCGA-LIHC (https://portal.gdc.cancer.gov/projects/TCGA-LIHC, accessed on 20 December 2024). We retrieved clinical data for HCC patients from TCGA, excluding those with missing survival data or an OS of <30 days. Ensembl IDs for lncRNAs, protein-coding genes, and miRNAs were mapped to their official gene symbols. The triaptosis-related gene set, comprising 14 genes, was adopted from a previous study (Table 1) [9]. scRNA-seq datasets of HCC and matched normal liver samples were collected from the Gene Expression Omnibus (GEO; GSE242889: http://www.ncbi.nlm.nih.gov/geo/, accessed on 24 December 2024) [27].

### 4.2. Cell Culture and Reagents

The human hepatocellular carcinoma (HCC) cell lines Huh7 and HCCLM3 were acquired from the Cell Bank of the Chinese Academy of Sciences (Shanghai, China). These cells were routinely cultured in Dulbecco’s Modified Eagle Medium (DMEM; Gibco, Waltham, MA, USA), which was supplemented with 10% fetal bovine serum (FBS; Gibco, USA). The cells were kept in a humidified incubator at 37 °C with 5% CO_2_.

The inhibitors used in this study, including Z-VAD-FMK (ZVF, S7023), ferrostatin-1 (Fer-1, S7243), necrostatin-1 (Nec-1, S8037), and N-acetyl cysteine (NAC, S5804), were procured from Selleck. Their respective working concentrations were as follows: 30 μM for ZVF, 10 μM for Fer-1, 20 μM for Nec-1, and 1 mM for NAC. Menadione sodium bisulfite (MSB; M2518, Sigma-Aldrich, St. Louis, MO, USA) was dissolved in sterile water and used at final concentrations of 0, 12.5, 25, and 50 μM for 12 h unless otherwise specified.

### 4.3. Cell Viability Assay

Following adhesion for 24 h in 96-well plates (3000 Huh7 cells/well), the cells were treated with increasing doses of MSB (0, 12.5, 25, 50 μM). Subsequently, 100 μL of fresh medium with 10% CCK-8 reagent (MA0218; Meilunbio, Dalian, China) was introduced into each well. After a 2 h incubation at 37 °C, absorbance was quantified at 450 nm using a Multiskan Spectrum 1500 spectrophotometer (Thermo Fisher Scientific, Waltham, MA, USA).

### 4.4. Establishment of Hepatoma Xenograft Model

Hepatoma xenograft models were established in male BALB/c nude mice (n = 5; 4 weeks old, ~20 g, purchased from Shanghai SLAC Laboratory Animal Co., Ltd., Shanghai, China). All animal experiments were performed in accordance with the guidelines approved by the Institutional Animal Care and Use Committee of Sir Run Run Shaw Hospital (SRRSH), with mice maintained in the pathogen-free facility of the hospital’s Laboratory Animal Research Center. To initiate tumor growth, 1 × 10^7^ Huh7 cells suspended in 200 μL PBS were injected subcutaneously into the right flank of each mouse. Tumor volume was monitored every 48 h and calculated using the following formula: V = (L × W^2^)/2, where L and W denote the longest and shortest diameters, respectively. Mice (n = 5 per group) received MSB (150 μg/mL) in drinking water for two weeks beginning 24 h after tumor cell inoculation; control mice received vehicle only. Mice were euthanized two weeks post-injection, and tumors were harvested, weighed, and imaged.

### 4.5. ROS and TUNEL Staining

To assess intracellular ROS levels, cells were loaded with 10 μM DCFH-DA (DCFH-DA; #S0035S, Beyotime, Shanghai, China) in serum-free medium for 30 min at 37 °C in darkness. Rosup (#S0035S-2, Beyotime, China), a proprietary compound mixture used as a positive control inducer of intracellular ROS, was applied at a concentration of 50 μg/mL for 30 min. Following three washes with PBS, the fluorescent signals were visualized under a Leica DM2500 fluorescence microscope (Germany), and the proportion of ROS-positive cells was quantified using ImageJ software (v1.53).

For the detection of apoptosis in tumor tissues, 5 μm paraffin-embedded sections were processed through deparaffinization, rehydration, and permeabilization with 20 μg/mL Proteinase K for 15 min at room temperature. **Apoptotic** cells were subsequently labeled employing the TUNEL assay kit (C1089, Beyotime, China). After counterstaining cell nuclei with DAPI (1 μg/mL; D9542, Sigma-Aldrich), images were acquired with the same Leica DM2500 fluorescence microscope.

### 4.6. Data Pre-Processing, Filtering and Normalization

The scRNA-seq dataset was processed with Seurat (v4). Following the creation of a Seurat object, quality control was performed by excluding cells that contained fewer than 500 genes or where mitochondrial genes constituted over 30% of the total transcript count (Figure S2A–F) [27]. Gene expression was then log-normalized and scaled. We subsequently identified the top 2000 highly variable genes for downstream analysis. To integrate data and mitigate batch effects, we applied the Harmony algorithm (v1.2.1), which preserves biological heterogeneity while correcting for technical variance.

### 4.7. Clustering Division and Cell Type Annotation

Dimensionality reduction was performed by applying principal component analysis (PCA) to the highly variable genes (HVGs). The first 30 principal components were then utilized to construct a shared nearest neighbor (SNN) graph, which was subsequently clustered using the Louvain algorithm. Finally, the resulting cell clusters were visualized in two dimensions with t-distributed stochastic neighbor embedding (t-SNE) (Figure S2G). For subcluster analysis, the clustree package was used to select the resolution and 0.3 was chosen. FindAllMarkers function was utilized to find genes with differential expressions. The major cell types were identified based on typical cell type markers. Eventually, malignant cells, DCs, T/NK cells, Kupffer cells, proliferation cells, central venous LSECs, monocytes, hepatic progenitor cells, plasma cells, B cells, neutrophil cells, memory B cells, periportal LSECs, and mast cells were labeled (Figure S2H). Following the same processing pipeline with a resolution of 0.4, we identified 9 malignant cell subclusters and evaluated their tumor-related pathway activities using the AddModuleScore package.

### 4.8. Identification of Differentially Expressed lncRNAs (DELs) and Triaptosis Co-Expressed lncRNAs (TCLs)

Differentially expressed lncRNAs (DELs) between HCC and normal liver tissues were screened using the ‘limma’ R package (Version: limma_3.65.3), with stringent thresholds set at |log_2_(fold change)| > 1 and an adjusted *p* < 0.001. Subsequently, we constructed mRNA-lncRNA co-expression networks by performing Pearson correlation analysis on TCGA HCC RNA-seq data, focusing on the 12 triaptosis-related genes (TRGs). Transcript correlation links (TCLs) were identified based on a correlation coefficient (R) > 0.3 and a statistical significance of *p* < 0.001.

### 4.9. Prognostic lncRNA Screening via Univariate Cox Analysis

The overlapping lncRNAs derived from the DELs and TCLs were further screened for prognostic significance. This was accomplished through univariate Cox regression analysis, implemented with the ‘survival’ R package. LncRNAs significantly associated with overall survival (*p* < 0.05) were ultimately defined as triaptosis-related prognostic lncRNAs (TRLs) in HCC.

### 4.10. Triaptosis-Related Prognostic Signature Construction and Validation

A prognostic signature was developed using LASSO Cox regression (Via the glmnet R package) to identify the most predictive TRLs and compute their respective regression coefficients [28]. Employing tenfold cross-validation (minimum λ criteria) yielded a final set of five TRLs for model construction. Each patient’s risk score was computed as the sum of the product of each TRL’s coefficient and its expression level. Using the median score as the cutoff, patients were classified into low- and high-risk cohorts. The model’s performance was rigorously validated by time-dependent ROC analysis, Kaplan–Meier survival curves, and univariate and multivariate Cox regression.

### 4.11. Nomogram Development

A predictive nomogram incorporating the risk score and relevant clinicopathological variables was constructed with the R rms package to estimate the probability of OS at 1, 3, and 5 years. Calibration curves were then plotted to evaluate the agreement between the nomogram’s predictions and the actual observed survival outcomes [29].

### 4.12. Lentiviral Knockdown and Transfection

*LINC01134*-targeting shRNAs (sh*LINC01134*-1: 5′-GCTCCAGAACTACATCAAAT-3′; sh*LINC01134*-2: 5′-GCAAGACCTGAAGTGCTATAA-3′) and scrambled control shRNA were cloned into the pLKO.1 plasmid. Production of lentiviral particles involved co-transfection of HEK293T cells with psPAX2/pMD2.G packaging plasmids using Lipofectamine™ 3000 (L3000015; Thermo Fisher, Waltham, MA, USA). Following collection and 0.22 μm filtration of the supernatant at 48 h, the virus was concentrated via ultracentrifugation for subsequent transduction of HCC cells. Stable clones were then obtained under 1 μg/mL puromycin selection for 48 h.

### 4.13. Human Samples Acquisition

Freshly paired samples of tumor and adjacent normal liver tissues (n = 16) were acquired from HCC patients who underwent surgical resection at Sir Run Run Shaw Hospital, Zhejiang University School of Medicine (Hangzhou, China). The Ethics Committee of the hospital granted approval for this study (ethical code: 20210729-282), which was conducted in strict accordance with the ethical principles of the Declaration of Helsinki.

### 4.14. RNA Extraction and Quantitative Real-Time PCR

Total RNA was isolated from cultured cells with the RNA-Quick Purification Kit (AG21023, Accurate Biology, Wuhan, China). The purified RNA was then reverse-transcribed into cDNA using the Eco M-MLV RT Premix (AG11706, Accurate Biology). Quantitative real-time PCR was carried out on a QuantStudio 1 system (Thermo Fisher Scientific, Waltham, MA, USA) with SYBR Green master mix (AG11701, Accurate Biology). The thermal cycling protocol included an initial denaturation at 95 °C for 5 min, followed by 40 cycles of 95 °C for 10 s and 60 °C for 30 s. Gene expression was quantified using the 2^−ΔΔCt^ method, with *GAPDH* serving as the internal reference control. All primer sequences are listed in Table 2.

### 4.15. Cell Colony Formation Assay

To assess the clonogenic survival and proliferative capacity of single cells, a colony formation assay was performed. Briefly, Huh7 and HCCLM3 cells in the logarithmic growth phase were harvested by trypsinization to generate a single-cell suspension. After cell counting, the suspension was seeded at a low density (500 cells per dish) into 60 mm culture dishes containing complete medium and gently swirled to ensure uniform distribution. The cells were cultured at 37 °C under 5% CO_2_ for 14 days, with the medium replaced every 3 days. When visible colonies had formed, the culture medium was aspirated, and the cells were carefully rinsed twice with PBS. Colonies were fixed with 4% paraformaldehyde for 15 min and then stained with 0.1% crystal violet for 30 min. Excess stain was removed by gentle rinsing under running water. After air-drying, colonies were counted manually. All experiments were independently repeated three times. Statistical significance between groups was analyzed using Student’s *t*-test, with *p* < 0.05 considered statistically significant.

### 4.16. Wound Healing Assay

To assess cell migration, a wound healing assay was conducted. Cells were seeded into a 12-well plate and cultured until they formed a 100% confluent monolayer. A sterile 200 μL pipette tip was used to create a straight scratch through the cell layer, perpendicular to a reference line on the back of the plate. The detached cells were removed by gently washing three times with PBS, and the medium was replaced with a serum-free one. Images of the scratch were captured at 0 h and at specific time points thereafter (e.g., 24, 48 h) using an inverted microscope at the same location. The width of the scratch was measured using ImageJ software, and the cell migration rate was calculated by comparing the wound area at different time points (n = 3).

### 4.17. Transwell Migration and Invasion Assay

Huh7 and HCCLM3 cells (3 × 10^4^/well) in 100 μL serum-free DMEM were seeded into the upper compartments of 24-well transwell plates. For invasion assays, inserts were pre-coated with 50 μL 10% Matrigel (40183ES08, Yeasen, Shanghai, China). The lower compartment of the transwell chamber was filled with DMEM supplemented with 10% FBS to serve as a chemoattractant. Following a 24 h incubation period under standard conditions (37 °C, 5% CO_2_), the cells that had migrated or invaded to the underside of the membrane were fixed with 4% paraformaldehyde for 30 min and then stained with a 1% crystal violet solution for 20 min. The stained cells were finally visualized and quantified under a bright-field microscope. The number of cells in three random fields per membrane was counted using ImageJ software (v1.53).

### 4.18. Hematoxylin-Eosin (H&E) Staining and Immunohistochemistry (IHC)

Paraffin-embedded tumor tissues were sectioned into 5 μm slices for both H&E and IHC staining. Following deparaffinization and rehydration, H&E staining was performed to evaluate general histology.

For IHC, tissue sections were subjected to heat-induced antigen retrieval in citrate buffer (pH 6.0). After blocking endogenous peroxidases and non-specific sites, the sections were incubated overnight at 4 °C with an anti-Ki-67 antibody (1:500; A20018, Abclonal, Wuhan, China). This was followed by signal development with a secondary antibody and DAB substrate, with expression levels being quantified microscopically.

### 4.19. Western-Blot Analysis

Proteins were isolated from Huh7 cells using RIPA lysis buffer containing phosphatase inhibitors, and protein concentrations were quantified with a BCA assay kit (P0012, Beyotime, Shanghai, China). Equal amounts of protein (20 µg per lane) were separated by SDS-PAGE on 12% gels at 120 V for 60 min and subsequently transferred to a PVDF membrane at 300 mA for 120 min. The membrane was blocked with 5% non-fat milk in TBST for 1 h at room temperature, followed by incubation with primary antibodies against MTM1 (1:1000, #13924-1-AP, Proteintech, Wuhan, China) and GAPDH (1:1000, #60004-1-Ig, Proteintech, Wuhan, China) overnight at 4 °C. After washing, the membrane was incubated with an HRP-conjugated secondary antibody (1:10000, #SA00001-2, Proteintech, Wuhan, China) for 1 h at room temperature. Protein bands were visualized using an enhanced chemiluminescence (ECL) detection system (Thermo Fisher, Waltham, MA, USA), and band intensities were analyzed using Image Lab software (version 6.1).

### 4.20. Statistical Analysis

All statistical analyses were performed using R software (version 4.1.2) or GraphPad Prism (Version 9.0.0), with a two-sided *p*-value < 0.05 considered statistically significant. Specific statistical methods applied in this study are detailed as follows:

#### 4.20.1. Differential Expression and Clinical Feature Analysis

The Mann–Whitney U test was used to compare lncRNA and gene expression levels between unpaired HCC and normal liver tissues from the TCGA-LIHC cohort. The paired *t*-test was applied for comparisons of gene expression in our in-house cohort of matched tumor and adjacent normal tissues (n = 16). The chi-square test was employed to analyze the associations between the TRL risk signature and categorical clinicopathological variables (e.g., gender, tumor grade, stage).

#### 4.20.2. Group Comparisons in Functional Experiments

For comparisons between two groups of continuous data (e.g., CCK-8 assays, transwell cell counts, tumor volume/weight in vivo, ROS fluorescence intensity, qRT-PCR, and Western blot quantification), the unpaired two-tailed Student’s *t*-test was used. For comparisons among three or more groups (e.g., dose–response curves to MSB, rescue experiments with cell death inhibitors), one-way analysis of variance (ANOVA) followed by an appropriate post hoc test (e.g., Tukey’s test) was applied. Data are presented as mean ± standard deviation (SD) from at least three independent experiments.

## 5. Conclusions

In summary, this study bridges triaptosis and lncRNA-mediated oncogenesis and development in HCC. By delineating the *LINC01134*-*MTM1*-PI(3)P axis, we provide a mechanistic foundation for exploiting triaptosis regulatory networks in HCC therapy. The 5-lncRNA prognostic signature offers a robust tool for risk stratification, and *LINC01134* emerges as a dual biomarker and therapeutic target. These findings advance precision oncology by integrating molecular insights with clinical applications, paving the way for innovative strategies to combat this recalcitrant malignancy.

## Figures and Tables

**Table 1:** Triaptosis-related genes.

Official Symbol	Official Full Name
*PIK3R4*	Phosphoinositide-3-Kinase Regulatory Subunit 4
*MTM1*	Myotubularin 1
*SH3GL3*	SH3 Domain Containing GRB2 Like 3
*ELMO2*	Engulfment And Cell Motility 2
*NCKAP1*	NCK-Associated Protein 1
*ACTN2*	Actinin Alpha 2
*KXD1*	KxDL Motif Containing 1
*WDR91*	WD Repeat Domain 91
*FYCO1*	FYVE and Coiled-Coil Domain Autophagy Adaptor 1
*RAB9A*	RAB9A, Member RAS Oncogene Family
*SCARB2*	Scavenger Receptor Class B Member 2
*ANXA8*	Annexin A8
*CLCN4*	Chloride Voltage-Gated Channel 4
*ATP13A2*	ATPase Cation Transporting 13A2

**Table 2:** Primers for RT-qPCR in this study.

Primer Name	Sequence (5′-3′)
*HPN-AS1-F*	CACGTCCCTTCCGTCTTGTC
*HPN-AS1-R*	CACGTCTACACCACATGGCT
*DDX11-AS1-F*	TGCTACTGTGGAGGACGTTG
*DDX11-AS1-R*	ACGTGCAGTCTTCTGAGTCC
*LINC01134-F*	CAACACCCTACCTGGCTCTG
*LINC01134-R*	CTGGGAGTGCGGACAGAAAT
*AC009283.1-F*	GCATCTGAGCAGCTGTGCAGCA
*AC009283.1-R*	CCTCCTCATCATCCTCCTGTGGGT
*GAPDH-F*	CTCTGCTCCTCCTGTTCGAC
*GAPDH-R*	ACCAAATCCGTTGACTCCGA
*MTM1-F*	GGCCCCATTAAGGGAAGAGTT
*MTM1-R*	CTTGTCGCGCCTCCCATTT
*AC009005.1-F*	GGCAAACATCTCTTGTCCATCCT
*AC009005.1-R*	CTCTCCGCATATCCCTCCTTCT

## Data Availability

The datasets that support the conclusions of this article are included within the paper and its accompanying supplementary Files. Any additional information required is available from the corresponding authors upon reasonable request. The analysis methods and packages used are described in Section 4. All other R codes and analyses are available from the corresponding authors upon request.

## Supplementary Materials

The following supporting information can be downloaded at https://www.mdpi.com/article/10.3390/ph18111691/s1. Figure S1. Transcriptomic profiling of Huh7 cells following MSB treatment. Figure S2. Data processing and cell type annotation. Figure S3. Dimensionality reduction analysis comparing high- and low-risk groups in the TCGA cohort. Figure S4. Differential expression of the 5-lncRNA prognostic signature in HCC versus adjacent normal tissues. Table S1: Cluster-specific signature markers identified in the single-cell RNA sequencing analysis. Table S2: Differentially expressed lncRNAs between HCC and normal tissues. Table S3: Correlation between triaptosis-related genes and lncRNAs. Table S4: Unicox analysis was performed to obtain prognosis-related lncRNAs with HCC patients. Table S5: Lasso analysis was performed to obtain 5 lncRNAs signature with HCC patients from TCGA cohorts.

## Abbreviations

HCC	Hepatocellular carcinoma
LncRNAs	Long non-coding RNAs
MSB	Menadione sodium bisulfite
ROS	Reactive oxygen species
TCGA	The Cancer Genome Atlas
FPKM	Fragments per kilobase of transcript per million mapped reads
TRGs	Triaptosis-related genes
TRLs	Triaptosis-related lncRNAs
DELs	Differentially expressed lncRNAs
TCDELs	Triaptosis-Correlated Differentially Expressed lncRNAs
PCA	Principal Component Analysis
t-SNE	t-Distributed Stochastic Neighbor Embedding
OS	Overall survival
Fer-1	Ferrostatin-1
Nec-1	Necrostatin-1
ZVF	Z-VAD-FMK
NAC	N-acetyl cysteine
H&E	Hematoxylin–eosin
IHC	Immunohistochemistry
DCFH-DA	2,7-dichlorodihydrofluorescein diacetate
LASSO	Least absolute shrinkage and selection operator
GSEA	Gene set enrichment analysis
KEGG	Kyoto Encyclopedia of Genes and Genomes
FBS	Fetal bovine serum
SRRSH	Sir Run Run Shaw Hospital
GAPDH	Glyceraldehyde 3-phosphate dehydrogenase
AUC	Area under the curve
RIPA	Radioimmunoprecipitation assay
scRNA-seq	Single-cell RNA sequencing
GEO	Gene Expression Omnibus
DCs	Dendritic cells
LSECs	Liver sinusoidal endothelial cells
ROC	Receiver operating characteristic
FFPE	Formalin-fixed paraffin-embedded
