# Supplementary material for: From Single-Cell and Bulk Transcriptomic Integration to Functional Verification: Triaptosis-Associated lncRNA Signature Predicts Survival and Guides Therapy in Hepatocellular Carcinoma
Source: Pharmaceuticals (Basel). 2025 Nov 7;18(11):1691. doi: 10.3390/ph18111691 (PMC12655504; doi:10.3390/ph18111691)
Supplement: Supplementary file 1 [file pharmaceuticals-18-01691-s001.zip › supplemental figures.pdf]

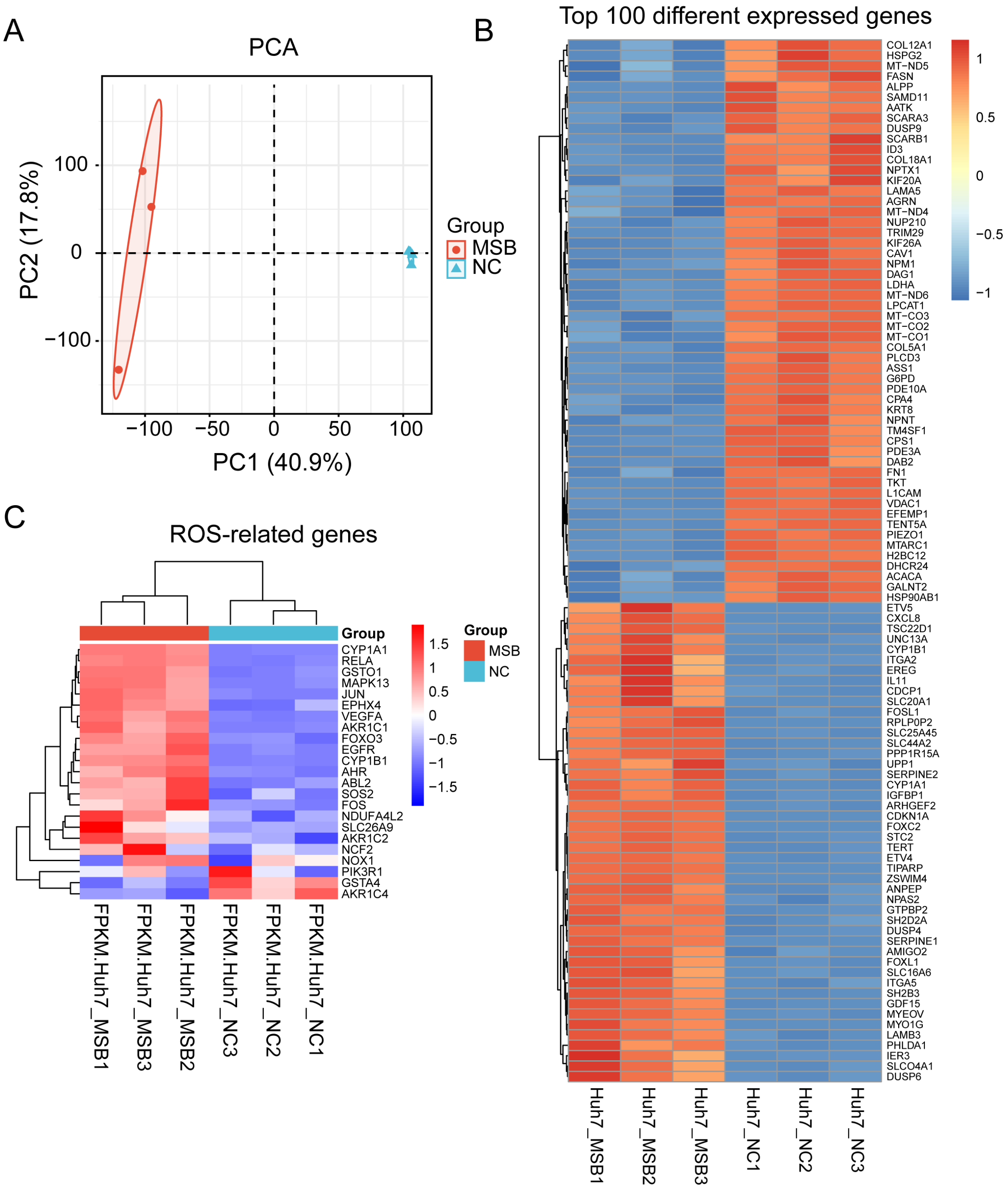

Figure S1. Transcriptomic profiling of Huh7 cells following MSB treatment.
(A) Principal component analysis (PCA) of Huh7-MSB and Huh7-NC groups, n=3. (B) Heatmap of the top 100 most significantly differentially expressed genes from the transcriptome sequencing of Huh7-MSB versus Huh7-NC groups. (C) Heatmap of differentially expressed genes enriched in the ROS pathway between Huh7-MSB and Huh7-NC groups.

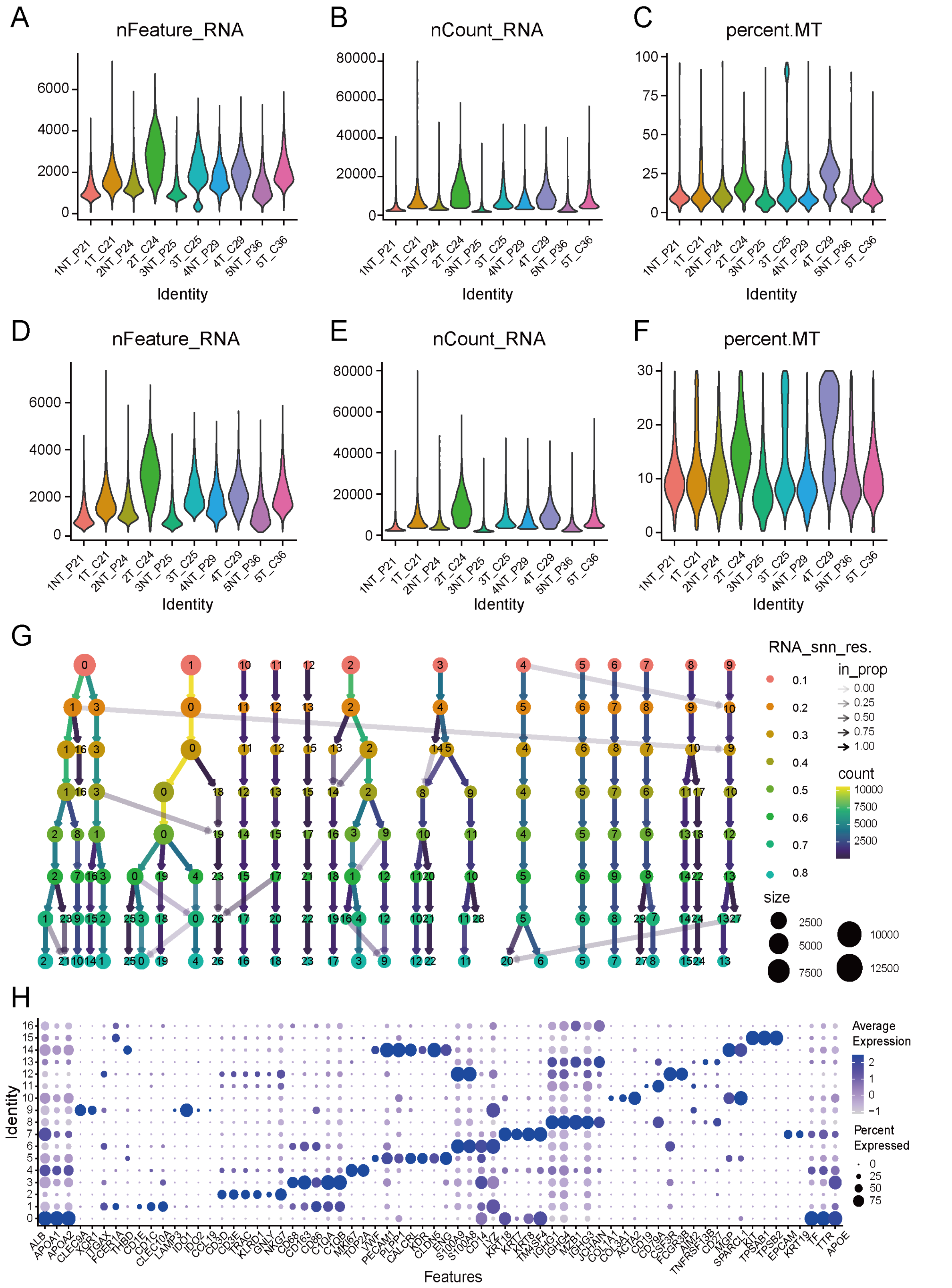

Figure S2. Data processing and cell type annotation.

A–C. Violin plots displaying pre-quality control (QC) metrics for each sample: (A) number of detected genes per cell (*nFeature RNA*), (B) total UMIs per cell (*nCount RNA*), and (C) percentage of mitochondrial gene expression (*percent MT*). D–F. Violin plots showing post-QC metrics for each sample: (D) *nFeature RNA*, (E) *nCount RNA*, and (F) *percent MT*. G. Clustree plot illustrating unsupervised clustering stability across varying resolution parameters. H. Dot plot representing expression levels of marker genes used to annotate major cell types.

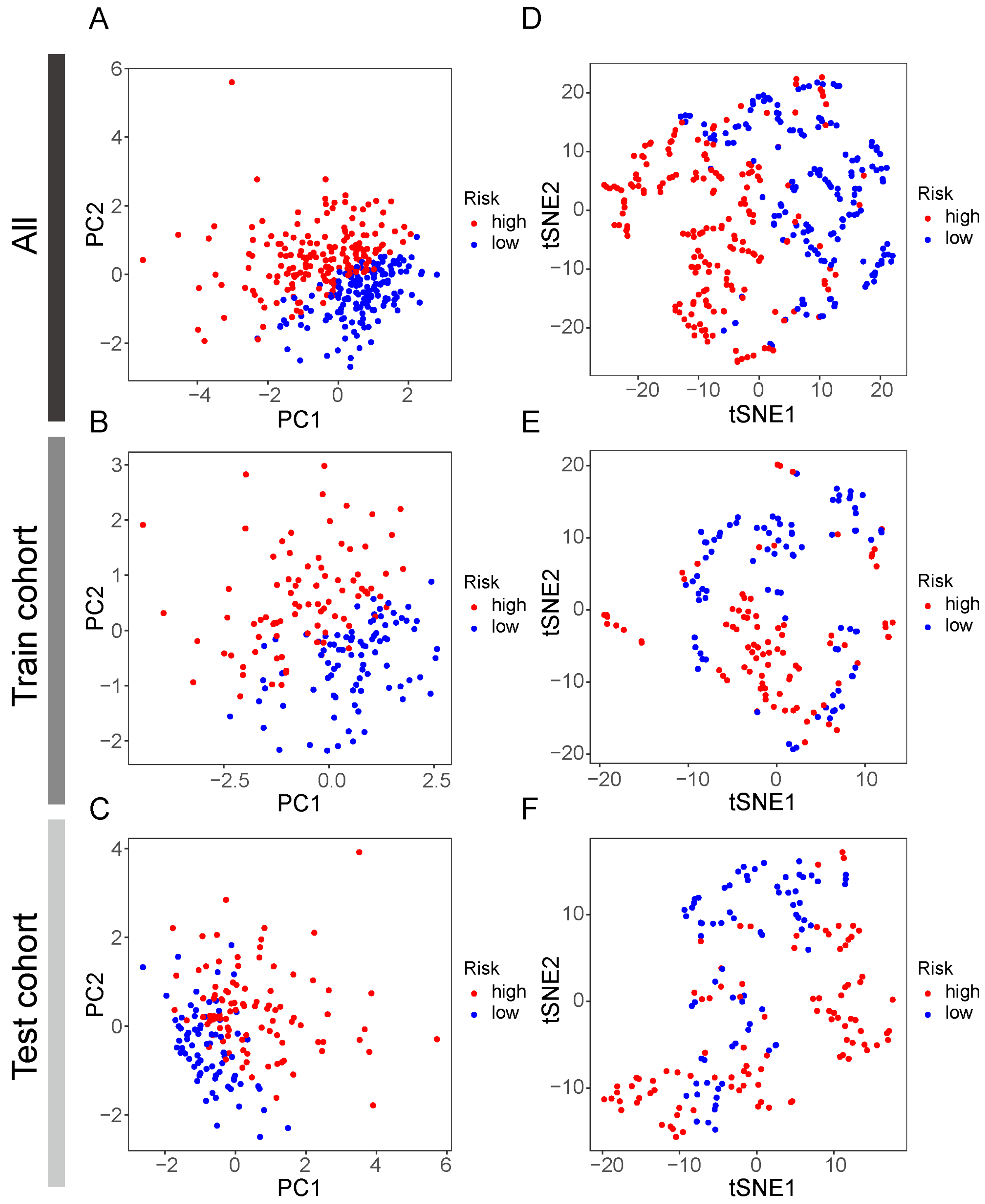

Figure S3. Dimensionality reduction analysis comparing high- and low-risk groups in the TCGA cohort.

A–C. Principal component analysis (PCA) of the (A) entire cohort, (B) training cohort, and (C) test cohort. D–F. t-distributed stochastic neighbor embedding (t-SNE) analysis of the (D) entire cohort, (E) training cohort, and (F) test cohort.

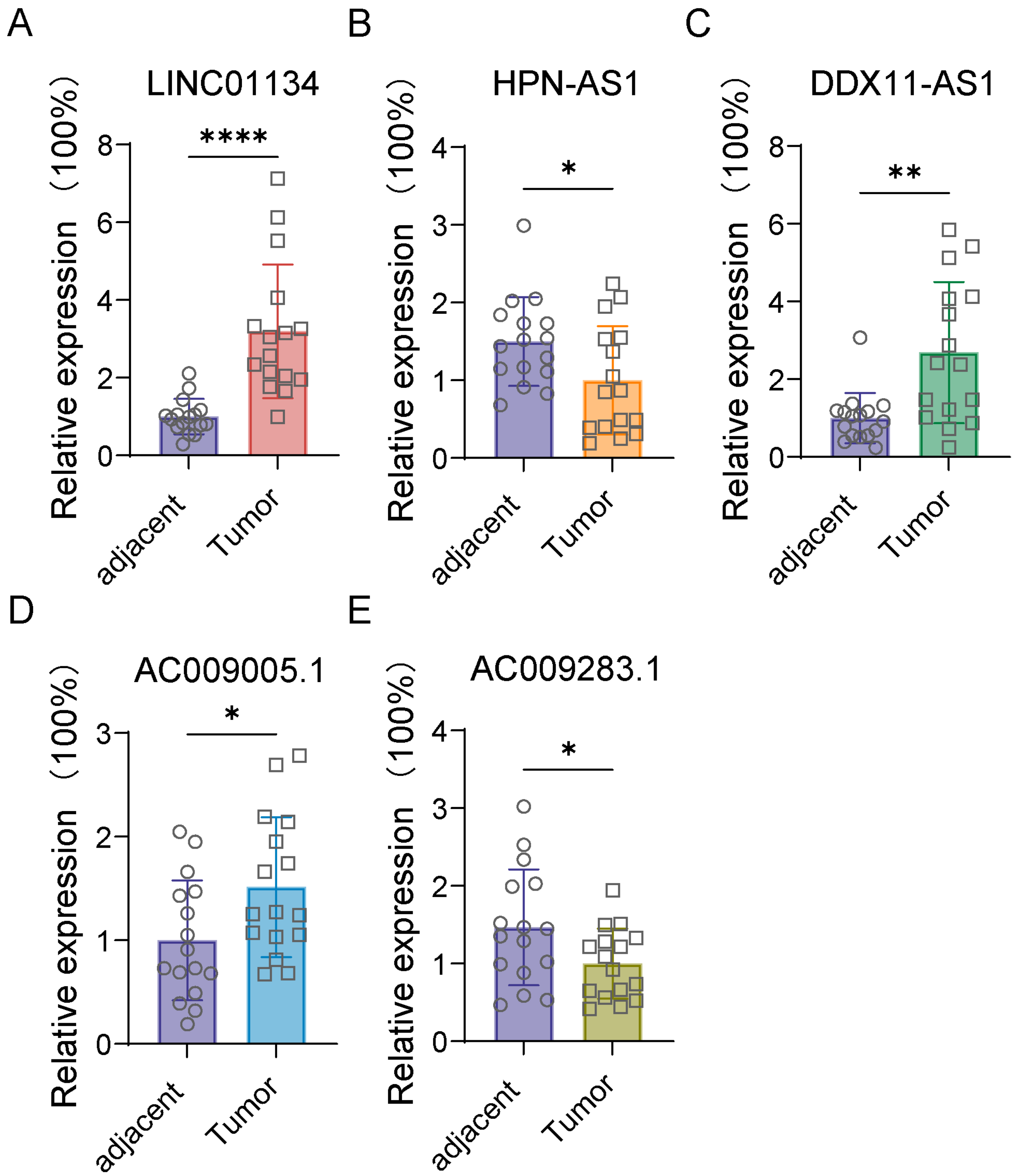

Figure S4. Differential expression of the 5-lncRNA prognostic signature in HCC versus adjacent normal tissues.

A–E. Relative expression levels of (A) *LINC01134*, (B) *HPN-AS1*, (C) *DDX11-AS1*, (D) *AC009005.1*, and (E) *AC009283.1* in HCC tissues compared to normal controls. Statistical significance was assessed using unpaired Student's t-test (*P < 0.05, **P < 0.01, ****P < 0.0001).
